# Assessing the applicability of ^19^F labeled tryptophan residues to quantify protein dynamics

**DOI:** 10.1007/s10858-022-00411-2

**Published:** 2023-01-14

**Authors:** Christina Krempl, Remco Sprangers

**Affiliations:** Department of Biophysics I, Regensburg Center for Biochemistry, University of Regensburg, 93053 Regensburg, Germany

**Keywords:** Fluorine NMR, Protein dynamics, CPMG relaxation dispersion, Conformational exchange, KIX domain, Dcp1, Dcp2, DcpS, mRNA decapping

## Abstract

Nuclear magnetic resonance (NMR) spectroscopy is uniquely suited to study the dynamics of biomolecules in solution. Most NMR studies exploit the spins of proton, carbon and nitrogen isotopes, as these atoms are highly abundant in proteins and nucleic acids. As an alternative and complementary approach, fluorine atoms can be introduced into biomolecules at specific sites of interest. These labels can then be used as sensitive probes for biomolecular structure, dynamics or interactions. Here, we address if the replacement of tryptophan with 5-fluorotryptophan residues has an effect on the overall dynamics of proteins and if the introduced fluorine probe is able to accurately report on global exchange processes. For the four different model proteins (KIX, Dcp1, Dcp2 and DcpS) that we examined, we established that ^15^N CPMG relaxation dispersion or EXSY profiles are not affected by the 5-fluorotryptophan, indicating that this replacement of a proton with a fluorine has no effect on the protein motions. However, we found that the motions that the 5-fluorotryptophan reports on can be significantly faster than the backbone motions. This implies that care needs to be taken when interpreting fluorine relaxation data in terms of global protein motions. In summary, our results underscore the great potential of fluorine NMR methods, but also highlight potential pitfalls that need to be considered.

**Supplementary Information:**

The online version contains supplementary material available at 10.1007/s10858-022-00411-2.

## Introduction

Most proteins fold into specific three dimensional structures to perform their functions. These structures are not static but undergo a multitude of conformational changes at timescales that range from picoseconds to hours (Palmer 2004; Henzler-Wildman and Kern 2007). Especially for enzymes these structural rearrangements are important for function, however, unfortunately, our knowledge regarding these dynamics is often insufficient.

NMR spectroscopy is unique in its ability to detect and quantify molecular motions in biomolecules (Palmer 2004; Audin et al. 2013; Alderson and Kay 2021; Wurm et al. 2021). Especially biomolecular motions that occur on the same timescale as biological catalysis are of interest, as those conformational changes might be directly correlated with function. Turnover rates of enzymes range from roughly 1 to 10^5^ per second and depending on the populations of the conformational states involved, these motions can be quantified with Carr-Purcell-Meiboom-Gill (CPMG) (Farber and Mittermaier 2015) and rotating-frame (R_1ρ_) (Palmer and Massi 2006) relaxation dispersion (RD) experiments and/or exchange spectroscopy (EXSY) (Kloiber et al. 2011) and CEST approaches (Vallurupalli et al. 2017). From these NMR experiments thermodynamic (populations) and kinetic (exchange rates) information that is associated with a given exchange process in a biomolecule can be extracted. The majority of these experiments is based on the recording of a set of ^1^H-^15^N or ^1^H-^13^C correlation spectra, which often requires a significant amount of spectrometer time.

An alternative to these approaches is the use of the fluorine (^19^F) nucleus for NMR experiments. Fluorine is nearly completely absent from biological samples, but it can be artificially incorporated into proteins, e.g., through the use of fluor-tryptophan residues (Crowley et al. 2012; Lu et al. 2019), the incorporation of other fluorinated amino acids (Furter 1998; Khan et al. 2006; Jackson et al. 2007; Ycas et al. 2020) or through the attachment of fluorinated methyl groups (^12^C-^19^F_3_) to cysteine side-chains (Ye et al. 2015). In addition, ^19^F can be incorporated into nucleic acids through the use of selectively fluorinated nucleotides (Hennig et al. 2007; Puffer et al. 2009; Sochor et al. 2016; Chrominski et al. 2020). This targeted ^19^F labeling of biomolecules presents several advantages compared to conventional ^15^N or ^13^C labeling strategies. First, the limited number of fluorine nuclei in the biomolecule results in simple NMR spectra with only one or a few resonances. One dimensional (1D) spectra, that can be recorded relatively rapidly, thus suffice to obtain site-specific information. Second, the large chemical shift dispersion of ^19^F (Lau and Gerig 2000) can result in a large chemical shift difference between the ground and excited states of a protein that leads to large amplitudes in RD curves. Third, the costs for ^19^F labeling are low and deuteration of larger proteins is usually not required as the fluorine is often remote from protons in the complex. Finally, recent advances in NMR probe-head design allow for the tuning of the ^1^H channel to ^19^F, thereby bypassing the need for obtaining dedicated fluorine probe-heads. Taken together, ^19^F NMR methods provide a complementary approach to detect and quantify dynamic processes in biomolecules (Kitevski-LeBlanc and Prosser 2012; Gronenborn 2022). To that end, we recently introduced a number of ^19^F relaxation dispersion (RD) experiments that are able to quantify dynamic processes (Overbeck et al. 2020, 2022).

Here, we aim at addressing two questions that challenge the general applicability of ^19^F NMR methods to obtain biologically relevant insights into protein dynamics. For a number of model systems, we probe if motions in these proteins are affected by the incorporation of 5-fluorotryptophan residues, as a replacement of a proton with a fluorine atom can potentially influence global protein dynamics due to differences in electronegativity and van der Waals radii. Subsequently, we assess if the introduced ^19^F labels are reliable probes to report on global motions in these proteins and if ^19^F and ^15^N derived dynamic parameters are in agreement.

We focus on four model proteins: (i) the KIX domain of the transcriptional co-activator CREB binding protein (Wang et al. 2012), (ii) Dcp1 and (iii) Dcp2 that together form the Dcp1:Dcp2 mRNA decapping complex (Wurm and Sprangers 2019) and (iv) the DcpS scavenger decapping enzyme (Gu et al. 2004; Fuchs et al. 2020). These proteins have been shown to exhibit global dynamics on different timescales. Motions in the KIX domain take place in the medium fast millisecond time regime (k_ex_ ~ 550 s^−1^) (Brüschweiler et al. 2009). Dcp1 possesses an aromatic groove that exchanges between different states with an exchange rate around 1200 s^−1^ (Wurm et al. 2016). In the absence of an mRNA substrate, the mRNA decapping complex Dcp1:Dcp2 furthermore exchanges between two distinct conformations in the fast millisecond time regime (k_ex_ ~ 2800 s^−1^) (Wurm et al. 2017). The motion of the scavenger decapping enzyme DcpS takes place in the slow millisecond to seconds time regime, depending on the concentration of the substrate (k_ex_ = 1–20 s^−1^) (Neu et al. 2015). All these complexes contain at least a single tryptophan residue in a region that has been shown to be dynamic. Importantly, for the enzymes Dcp2 and DcpS the observed motions are important for the catalytic cycle.

Here, we show that the incorporation of 5-fluorotryptophan does not change the dynamics of the studied proteins as judged by ^15^N and methyl-TROSY studies. However, for the KIX domain, for Dcp1 and for Dcp2, the exchange parameters that we obtain from ^19^F RD measurements are not in agreement with those extracted from ^15^N RD experiments. The cause of these inconsistencies likely arises from faster motions of the tryptophan side chains. These fast dynamics result in CPMG RD dispersion profiles that are simultaneously influenced by side chain and backbone motions and that thus cannot be interpreted with a simple two-state exchange model. For the DcpS decapping enzyme, we show that the dynamics that we extract from ^19^F EXSY measurements are in excellent agreement with previous results that were based on methyl TROSY experiments (Tugarinov et al. 2003; Neu et al. 2015; Schütz and Sprangers 2019). Our findings thus highlight that it is possible, but potentially misleading, to draw conclusions on global protein dynamics based on ^19^F data derived from a limited number of probes.

## Material and methods

### Molecular biology

All proteins (KIX domain of the human CBP protein, UniProt ID Q92793, residues 321-407, internal ID 893; Dcp1 from *S. pombe*, UniProt ID Q9P805, residues 1-127, internal ID 48; Dcp2 from *S. pombe*, UniProt ID Q9P805, residues 1-95, internal ID 52, or residues 1-243, internal ID 223; DcpS (with the inactivating N258A mutation) from *C. thermophilum*, UniProt ID G0S8A3, internal ID 2326) in this study were over-expressed in and isolated from *E. coli* BL21(DE3) CodonPlus-RIL cells (Stratagene). To that end, plasmids were transformed into the cells that were subsequently grown overnight at 37 °C on LB agar plates in the presence of chloramphenicol and kanamycin or ampicillin. Single colonies were resuspended in a 25 ml LB pre-culture and grown to an optical density OD600 of 0.8–1.0. 1 ml of this culture was used to inoculate a 100 ml M9 minimal medium based pre-culture, that was incubated overnight at 37 °C. The overnight culture was finally diluted with 900 ml M9 medium and grown to an OD600 of 0.6 at 37 °C, after which 50 mg 5-fluoroindole (Crowley et al. 2012) that was dissolved in DMSO at a 100 mg/ml stock concentration was added. 1 h later the temperature was changed to 20 °C and protein expression was induced by addition of 1 mM IPTG. For ^15^ N labeling, ^15^NH_4_Cl was used as the sole nitrogen source, while isoleucine-δ_1_ and methionine-ε methyl group labeling was achieved by adding 50 mg/l α-ketobutyric acid and 100 mg/l of [methyl- ^13^CH_3_] methionine 1 h prior to induction. Cells were harvested 12–18 h after induction by centrifugation at 6000×*g* for 30 min.

Cell pellets were resuspended in buffer A (150 mM NaCl, 50 mM NaPB, pH 7.4, 5 mM imidazole), supplemented with 1:1000 Triton X-100, 1 mg/ml lysozyme and 1 mM EDTA and lysed by sonication. Then, 2 mM MgSO_4_ was added and the cell debris was removed by centrifugation at 18,000×*g* for 30 min. The supernatant was filtered with a 1.2 μm syringe filter and applied to a Ni–NTA gravity flow column that was equilibrated with buffer A. The column was washed with 5–10 column volumes of buffer A and the bound protein was eluted with buffer B (150 mM NaCl, 50 mM NaPB, pH 7.4, 300 mM imidazole). The His-tag was removed either by TEV cleavage during dialysis overnight against 25 mM Tris, pH 8.0, 75 mM NaCl, 1 mM DTT at 4 °C (Dcp1, Dcp2 and DcpS) or by incubation with Thrombin during dialysis overnight against 150 mM NaCl, 25 mM NaPB, pH 7.4, 1 mM DTT at 4 °C (KIX domain). The cleaved tag was separated from the target protein using a second Ni–NTA column that was equilibrated with buffer A. The column flow-through and wash fractions were collected and further purified by size exclusion chromatography using a 16/600 Superdex S200 or 16/600 Superdex S75 gel filtration column in buffer C (25 mM NaCl, 25 mM HEPES pH 8.0, 1 mM DTT) (DcpS) or in buffer D (125 mM NaCl, 25 mM HEPES, pH 7.3, 1 mM DTT) (Dcp1, Dcp2, KIX). The fractions containing the purified protein were combined and concentrated to a final volume of 500 μl. For KIX, the buffer was exchanged to 50 mM potassium phosphate buffer, pH 5.8, 25 mM NaCl. NMR samples were supplemented with 0.03% NaN_3_ and 5% D_2_O. Final protein concentrations varied between 100 and 500 μM.

### Assignments

Tryptophan resonances were assigned by a mutational approach that we often use to assign methyl group resonances (Sprangers et al. 2005; Sprangers and Kay 2007), where spectra of the wild-type complex were compared with spectra of the complex that lacked individual tryptophan residues. The W43A assignment mutation in Dcp2 residues 1-95 (internal ID 1070) and Dcp2 residues 1-243 (internal ID 1485) as well as the N258A (internal ID 2326), W77Y (internal ID 2277), W188Y (internal ID 2325), W220Y (internal ID 2360) and W208Y (internal ID 2361) mutations in DcpS were introduced using standard quick-change methods.

### NMR experiments

NMR spectra were recorded at 298 K (KIX) or 303 K (Dcp1, Dcp2, DcpS) on Bruker NEO spectrometers that operate at 500, 600 or 800 MHz proton frequency and that were equipped with nitrogen (500 and 600) or helium (800) cooled TCI probe heads and NEO consoles. ^19^F experiments were recorded at 500 or 600 MHz proton frequency by tuning the proton channel to the ^19^F frequency. Acoustic ringing artifacts were suppressed using a three pulse “aring” sequence (Overbeck et al. 2020). For ^15^N CPMG experiments frequencies (ω_CPMG_/2π) between 0 and 1000 Hz and relaxation delays of 50 ms (KIX) or 60 ms (Dcp1, Dcp2) were used. For ^19^F CPMG experiments frequencies (ω_CPMG_/2π) between 0 and 5000 Hz and a relaxation delay of 10 ms were used. Longitudinal exchange experiments were recorded using a series of 2 dimensional ^19^F-^19^F EXSY (NOESY) experiments on samples containing 0.4 mM DcpS (monomer concentration) in the presence of a 1:1, 1:2, 1:4, 1:6, 1:8, 1:10, 1:12 or 1:14 molar excess of m^7^GpppG using 19 different mixing times ranging from 3 μs to 800 ms. Due to fast relaxation of the ^19^F magnetization the acquisition time in the indirect dimension in the NOESY experiments was restricted to 2.8 ms. Errors in all experiments were extracted from two or three recorded sets of experiments. Methyl TROSY experiments were recorded using a SOFAST HMQC sequence (Schanda and Brutscher 2006), using a carbon acquisition time of 21 ms. NMR data were processed using the NMRPipe/NMRDraw software suite (Delaglio et al. 1995), figures displaying NMR spectra were produced using NMRView (onemoonscientific.com) and data were analyzed with in-house written Matlab scripts.

### Data analysis

For the CPMG experiments the effective transverse relaxation rates were calculated as R_2_^eff^ =  − (1/T_CPMG_)*ln(I/I_0_), where I is the intensity of the peak and I_0_ the reference intensity recorded without the constant time CPMG relaxation delay. Relaxation dispersion curves were numerically fitted to a two-state exchange model (Baldwin 2014), in which the populations (p_A_, p_B_ = 1 − p_A_), the exchange rate (k_ex_), the absolute value of the chemical shift difference between the two states ($\left| {\Delta \omega } \right|$) and the transverse relaxation rates (assumed to be identical for both states) were used as fitting parameters. Data from different residues and different magnetic fields were fitted simultaneously to a single exchange rate and a single population of the excited state. To extract the rates from the longitudinal exchange experiments the intensities of the cross- and autopeaks were fitted to one global two-state exchange process (Sprangers et al. 2005) in which I_cross_ = Ap_a_p_b_exp(− ρt_mix_)*[1 − exp(− k_ex_t_mix_)] and I_auto_ = Bexp(− ρt_mix_)*[p_a_^2^ + p_a_p_b_exp(− k_ex_t_mix_)]. Here, I_cross_ and I_auto_ are the intensity of the cross and auto peaks respectively, p_a_ and p_b_ are the populations for the open and the closed state of the enzyme that were fixed to 0.5 due to the symmetric nature of the dimer, ρ is the longitudinal relaxation rate, t_mix_ the mixing time, k_ex_ the exchange rate and A and B are scaling factors. To determine the goodness of the fits, Monte Carlo simulations with 100 fit iterations were used. In this process, the mean values of all data points were randomly varied according to a normal distribution with a width that corresponds to the respective standard deviation of the data point that was determined from multiple measurements.

## Results

### KIX domain of the CREB-binding protein

**Fig. 1:**
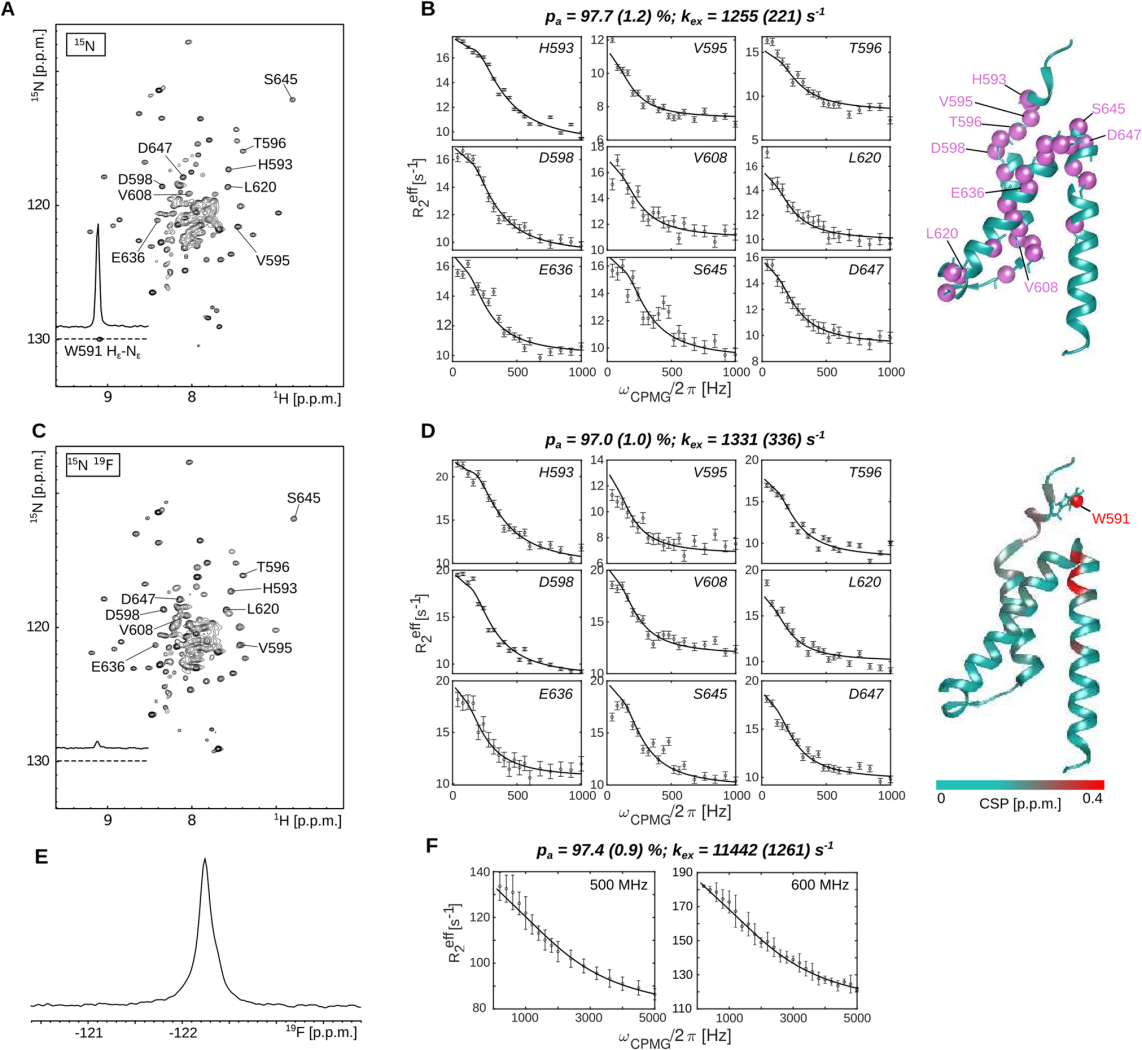
Influence of 5-fluorotrypthphan incorporation on the dynamics in the KIX domain. **A** ^1^H-^15^N spectrum of the ^15^N labeled KIX domain. The 1D trace in the spectrum shows the H_ε_-N_ε_ signal of the W591 side-chain at the N-terminal end of the domain. **B** ^15^N CPMG relaxation dispersion (RD) profiles that report on the folding-unfolding of the ^15^N labeled KIX domain. The residues that undergo chemical exchange are highlighted on the structure of the domain (Brüschweiler et al. 2013) (PDBid: 2LXT). **C** ^1^H-^15^N spectrum of the ^15^N, 5-fluorotryptophan labeled KIX domain. The strong reduction of the signal of the W591 H_ε_-N_ε_ side-chain resonance reveals the efficient incorporation of the ^19^F label. **D** The ^15^N CPMG RD profiles are not influenced by the incorporation of the 5-fluorotryptophan. The chemical shift perturbations (CSPs) that are induced by the ^19^F atom are limited to a region that is close to the tryptophan residue (Fig S2). **E** 1D ^19^F NMR spectrum of the 5-fluorotryptophan labeled KIX domain. **F** ^19^F CPMG RD profiles of the 5-fluorotryptophan labeled KIX domain, recorded at 500 and 600 MHz proton frequency

Dynamics in the KIX domain of the CREB-binding protein are important for the allosteric communication between two ligand binding sites (Brüschweiler et al. 2013). Extensive previous studies reveal that this domain exchanges between a folded and a partially unfolded high energy state with an exchange rate of ~ 550 s^−1^ (Brüschweiler et al. 2009). Here, we labeled the domain with ^15^N (Fig. 1A) and recorded nitrogen relaxation dispersion (RD) profiles (Figs. 1B, S1). Based on that data, we confirm that the domain undergoes a global two-site exchange process (p_a_ = 97.7 ± 1.2%, k_ex_ = 1255 ± 221 s^−1^). Those values deviate slightly from the previously reported exchange parameters, which we attribute to differences in the buffer composition. The KIX domain contains a single tryptophan residue that stacks on the top of the exchanging helical bundle and we reasoned that a partial unfolding of the KIX domain should be sensed by the aromatic side-chain. To validate that, we simultaneously labeled the KIX domain with ^15^N and 5-fluorotryptophan. The incorporation of the ^19^F label was highly efficient as can be judged from the strong reduction in the H_ε_-N_ε_ signal in the proton-nitrogen spectrum (Fig. 1C). Structurally, the impact of the ^19^F incorporation was minimal, as judged from the small CSPs that are limited to resonances of residues in close spatial proximity to the tryptophan residue (Fig. 1D; Fig S2). Subsequently, we recorded ^15^N CPMG RD experiments on the ^15^N labeled KIX domain that contains the 5-fluorotryptophan (Figs. 1D, S1, S2). A global fit of this data reveals that the exchange parameters (p_a_ = 97.0 ± 1.0%, k_ex_ = 1331 ± 336 s^−1^) were unaffected by the presence of the 5-fluorotryptophan. In line with these findings, it is possible to fit the ^15^N RD profiles of the ^15^N and the ^15^N-^19^F labeled samples simultaneously to one exchange process (p_a_ = 98.2 ± 0.4%; k_ex_ = 924 ± 136 s^−1^) (Figs. S3, S4). This demonstrates that the incorporation of the ^19^F label does not interfere with the global dynamics of the protein, which is a prerequisite for the applicability of ^19^F methods (Fig. 1E) to extract relevant information regarding protein motions. Next, we recorded ^19^F CPMG RD experiments at two field strengths and fitted these to a two-site exchange process (Fig. 1F). This analysis revealed a tenfold faster exchange rate (p_a_ = 97.4 ± 0.9%, k_ex_ = 11,442 ± 1261 s^−1^) compared to the ^15^N derived parameters (Fig. 1D). Attempts to simultaneously fit the ^15^N and ^19^F CPMG data to one exchange process were not successful (Fig. S5), which confirms that the motions that the ^19^F labeled tryptophan side chain experiences are different from the global backbone motions that we assessed using ^15^N RD experiments. A higher flexibility of the tryptophan side chain compared to the protein backbone is not unexpected and in that case the recorded RD curves will be a superposition of the slower backbone and the faster side chain motions. This results in data that can no longer be analyzed with a single two-state exchange model. This notion highlights the challenges and potential pitfalls that are associated with drawing conclusions regarding global protein motions based on ^19^F based measurements only.

### Dcp1:Dcp2 mRNA decapping complex

We next extended our analysis to the Dcp1:Dcp2 mRNA decapping complex that is composed of the decapping enzyme Dcp2 and its main activator Dcp1. Dcp2 consists of an N-terminal regulatory domain (NRD; amino acids 1-95) that interacts with Dcp1, a C-terminal catalytic NUDIX domain (CTD; amino acids 96-243) and a long C-terminal intrinsically disordered region (IDR; amino acids 244-743). Previous ^15^N based CPMG relaxation dispersion experiments revealed that Dcp1 contains a flexible groove that exchanges between two states at a rate of ~ 1200 s^−1^ and that can recruit proline-rich elements (Wurm et al. 2016). In addition, methyl based single quantum CPMG RD experiments on the Dcp1:Dcp2 complex showed that the NRD and CTD of Dcp2 undergo inter-domain motions at a rate of 2800 s^−1^ (Wurm et al. 2017). These large conformational changes are important for the regulation of the catalytic activity of the enzyme complex (Wurm and Sprangers 2019). Conveniently, it is possible to express Dcp1 and Dcp2 separately and fluorotryptophan labeling can thus be restricted to either Dcp1 or Dcp2.

**Fig. 2:**
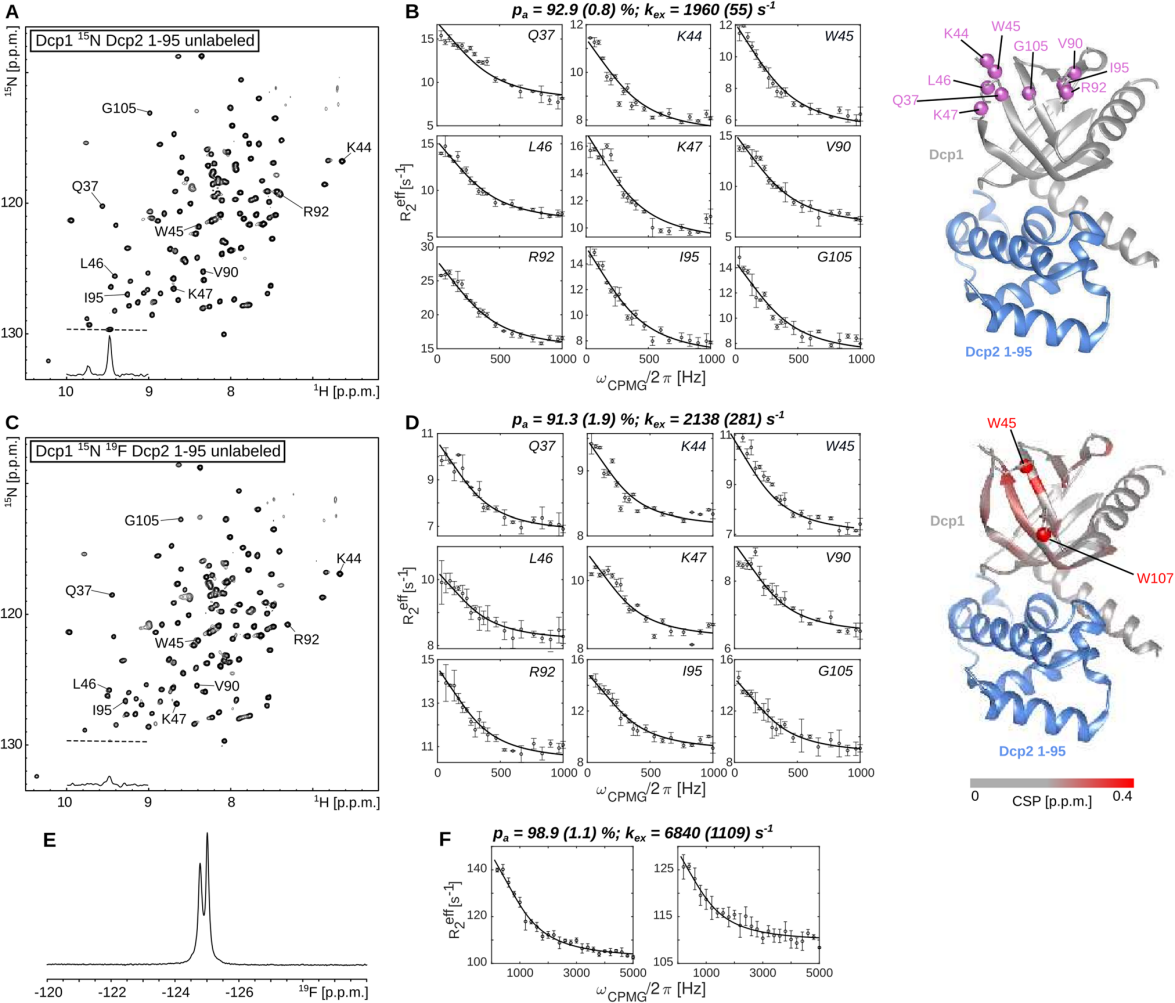
Influence of 5-fluorotrypthphan incorporation on the dynamics of Dcp1 in a minimal Dcp1:Dcp2 complex. **A** ^1^H-^15^N spectrum of the ^15^N labeled Dcp1 (Dcp2 is NMR inactive). The 1D trace in the spectrum shows one of the tryptophan H_ε_-N_ε_ signals. **B** ^15^N CPMG relaxation dispersion (RD) profiles that report on the dynamics in the aromatic groove of the Dcp1 protein (Wurm et al. 2016). The residues that undergo chemical exchange are highlighted on the structure of the domain (She et al. 2008) (PDBid: 2QKL). Dcp1 is colored gray, the Dcp2 regulator domain is NMR invisible and colored light blue. **C** ^1^H-^15^N spectrum of the ^15^N, 5-fluorotryptophan labeled Dcp1:Dcp2 complex. The strong reduction of the H_ε_-N_ε_ side-chain signal reveals the efficient incorporation of the ^19^F label. **D** The ^15^N CPMG RD profiles are not influenced by the incorporation of the 5-fluorotryptophan. The chemical shift perturbations (CSPs) that are induced by the two 5-fluorotryptophan residues are limited to the region that is close to the tryptophan residue (Fig S2). **E** 1D ^19^F NMR spectrum of the 5-fluorotryptophan Dcp1:Dcp2 complex. **F** ^19^F CPMG RD profiles of the upfield- and downfield ^19^F signals, recorded at 500 MHz proton frequency

We first prepared a minimal Dcp1:Dcp2 complex that contains ^15^N labeled Dcp1 and the unlabeled NRD of Dcp2 (Fig. 2A). ^15^N based relaxation dispersion experiments showed clear dynamics in the aromatic groove of Dcp1 (Fig. 2B, S6). Fitting these data to a global exchange process revealed a ground state population p_a_ of 92.9 ± 0.8% and an exchange rate k_ex_ of 1960 ± 55 s^−1^. These data agree with previous measurements, where small deviations in the exchange parameters are due to differences in the used buffer system. Subsequently, we prepared the Dcp1:Dcp2 complex but this time by labeling Dcp1 with ^15^N and 5-fluorotryptophan. The 5-fluorotryptophan labeling was efficient as judged by the strong reduction of the H_ε_-N_ε_ resonances in the 2D NMR spectrum (Fig. 2C). Replacement of the natural tryptophan residues with 5-fluorotryptophan resulted in minor chemical shift perturbations in the ^1^H-^15^N correlation map, revealing that the introduced fluorine atoms have no global effect on the structure of the complex (Figs. 2D, S7). We subsequently recorded ^15^N CPMG RD curves on this sample from which we extracted a ground state population of 91.3 ± 1.9% and an exchange rate k_ex_ of 2138 ± 281 s^−1^ (Fig. S6). Based on that, we conclude that the introduction of 5-fluorotryptophan had no measurable effect on the backbone dynamics of Dcp1. The absence of large differences in dynamics between the ^15^N and the ^15^N/^19^F labeled proteins can be further corroborated by the fact that it is possible to fit the nitrogen RD profiles from both datasets (without and with ^19^F labeling) simultaneously (Fig. S8), which results in a ground state population of 91.8 ± 1.1% and an exchange rate k_ex_ of 2220 ± 121 s^−1^ (Fig. S9).

The ^19^F NMR spectrum of the Dcp1 5-fluorotryptophan labeled Dcp1:Dcp2 complex shows two distinct resonances (Fig. 2E) that result from Trp 45 and 107 in Dcp1. We next recorded ^19^F CPMG RD experiments and extracted exchange parameters by jointly fitting the data of the two residues in the aromatic groove to a two-site exchange model. We obtained a population p_a_ of the ground state of 98.9 ± 1.1% and an exchange rate k_ex_ of 6840 ± 1109 s^−1^ (Fig. 2F). As observed for the KIX domain, this exchange rate is significantly faster than the rates obtained from ^15^N CPMG experiments, indicating that the ^19^F data does not report on the global exchange process that we quantified based on the ^15^N data. In agreement with that, it is not possible to fit the ^19^F and the ^15^N relaxation dispersion data simultaneously to one exchange process (Fig. S10). The faster timescale of the ^19^F data suggests that the fluorotryptophan side-chains are more flexible than the backbone of the protein and that the ^19^F tryptophan indole ring does not solely report on the global exchange process in the Dcp1 aromatic groove, although Trp 45 is centrally located in this groove.

**Fig. 3:**
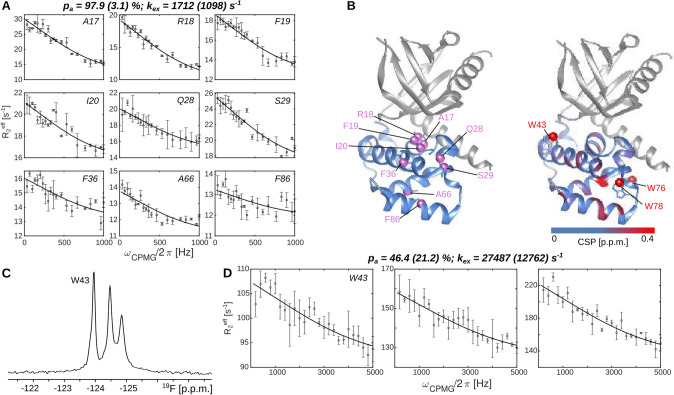
Measurement of dynamics in the Dcp2 N-terminal regulatory domain (NRD), in complex with Dcp1. **A** ^15^N CPMG relaxation dispersion (RD) profiles for a set of residues in the Dcp2 NRD. The data is recorded on a ^15^N, 5-fluorotryptophan labeled Dcp2 sample. **B** The observed dynamics cluster in the core of the Dcp2 NRD. Three tryptophan residues surround the mobile region. Dcp1 is NMR invisible and colored gray, the Dcp2 regulatory domain is colored light blue. The chemical shift perturbations (CSPs) that are induced by the three 5-fluorotryptophan residues in the Dcp2 domain are limited to the region that is close to the tryptophan residue (Fig. S2). **C** ^19^F NMR spectrum of the ^15^N, 5-fluorotryptophan labeled Dcp2 sample. The W43 resonance is assigned by mutagenesis. **D** ^19^F CPMG relaxation dispersion profiles of the three ^19^F resonances, recorded at 600 MHz proton frequency

In a next set of experiments, we assessed the motions in the Dcp2 NRD. To that end, we measured nitrogen RD experiments on a complex of unlabeled Dcp1 and ^15^N labeled NRD from Dcp2. The results indicate the presence of an exchange process in the Dcp2 NRD with a ground-state population of 97.9 ± 3.1% and an exchange rate k_ex_ of 1712 ± 1098 s^−1^ (Figs. 3A, S11). The three Dcp2 tryptophan residues are in close spatial proximity to this flexible region and thus potentially sense these global motions (Fig. 3A). To assess that, we labeled the Dcp2 NRD with 5-fluorotryptophan and obtained a fluorine spectrum with three distinct resonances (Fig. 3B). These three residues showed clear RD dispersions, however, the extracted exchange parameters (p_a_ = 52.1 ± 7.0%, k_ex_ = 17,795 ± 2967 s^−1^) reveal an exchange rate that was, as above, faster than the exchange rate that was extracted from the ^15^N data. As for KIX and Dcp1, it was hence not possible to fit the ^15^N and ^19^F RD data simultaneously (Fig. S12). Of note, the ^15^N-^19^F labeled Dcp2 protein expressed with yields that were sufficient for the recording of 1D ^19^F RD curves, but that were too low to allow for the recording of ^15^N relaxation data with sufficient signal to noise. We can thus not directly probe for potential changes in the backbone motions that could be caused by the ^19^F labeling. However, we anticipate that these changes are very small as the CSPs caused by the ^19^F introduction (Fig S13) are comparable to those that we observed upon introduction of ^19^F labels in the KIX domain and the Dcp1 protein.

**Fig. 4:**
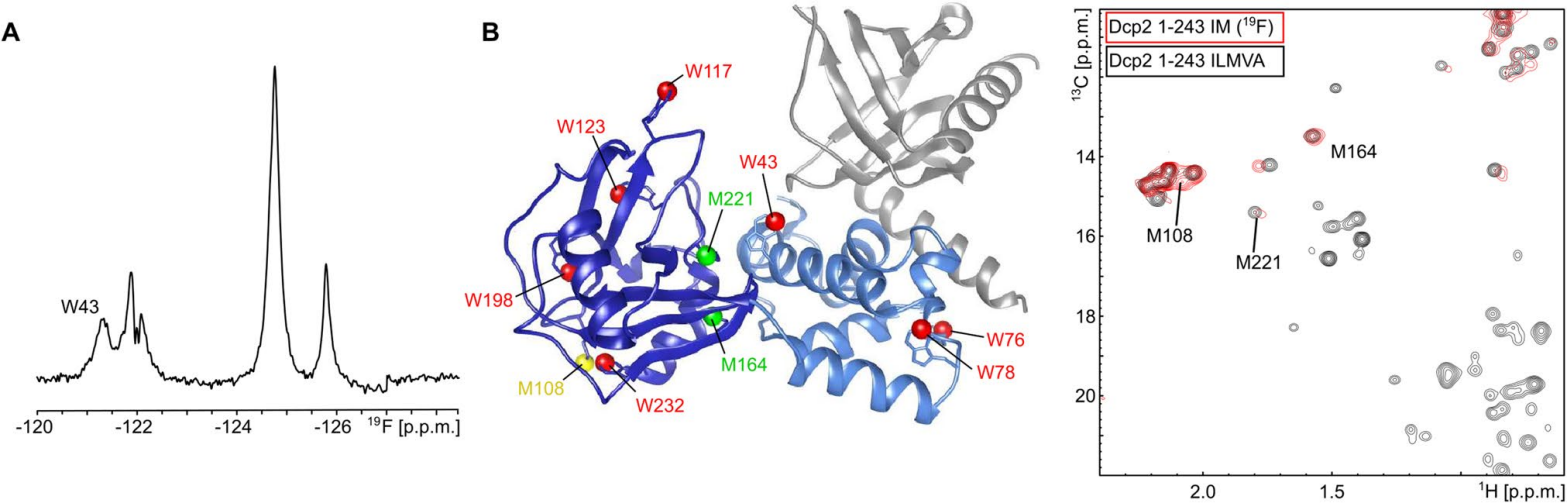
The Dcp2 regulatory (NRD) and catalytic domains (CTD), in complex with Dcp1. **A** The 1D ^19^F spectrum of the complex with 5-fluorotryptophan labeled Dcp2 reveals 5 resonances for the 7 tryptophan residues. The resonance of the gatekeeper tryptophan (W43) is assigned through mutagenesis. Note the shift of the W43 frequency between the complex with and without (Fig. 3B) the Dcp2 CTD. **B** Structure of the Dcp1-Dcp2 NRD-CTD complex. Dcp1 is NMR invisible and colored gray, the Dcp2 NRD is colored light blue and the Dcp2 CTD is colored dark blue. The seven tryptophan residues are colored red. Methionine 221 and 164, that report on the open-closed transitions in the complex, are colored green. Methionine 108, that experiences a CSP in the methyl TROSY spectrum due to the incorporation of 5-fluorotryptophan, is colored yellow. **C** Overlay of the methionine region of methyl TROSY spectra of the Dcp1 (NMR inactive): Dcp2 (ILMVA methyl labeled) complex (grey) and the Dcp1 (NMR inactive): Dcp2 (IM methyl and 5-fluorotryptophan labeled) complex (red). The methionine resonances of M221 and M164, that report on the conformation of the complex, are labeled

In the three examples above, we focused on relatively small assemblies, with molecular weights of 10 kDa (KIX domain) and 26 kDa (Dcp1:Dcp2-NRD). We next aimed at recapitulating the exchange between the open and closed state in the 46 kDa Dcp1:Dcp2-NRD-CTD complex (k_ex_ = 2800 s^−1^). In this process the CTD of Dcp2 can either dock onto the NRD of Dcp2 (closed conformation; 94%), or it can be detached from the NRD (open conformation; 6%) (Wurm et al. 2017). The Dcp2 NRD contains a catalytically important gatekeeper tryptophan residue (W43) (Floor et al. 2012) that is solvent exposed in the open conformation and tightly packed onto the Dcp2-CTD interface in the closed state (She et al. 2008). The exchange between these open and closed conformations of Dcp2 have been shown to be important for the regulation of the mRNA decapping activity (Wurm et al. 2017). To determine the ^19^F chemical shift of the “open” state of the gatekeeper 5-fluorotryptophan we first assigned W43 through mutagenesis in the Dcp1:Dcp2-NRD construct (Figs. 3B, S14), where the gatekeeper tryptophan is solvent exposed due to the lack of the CTD. Next, we prepared a Dcp1:Dcp2-NRD-CTD complex in which the seven Dcp2 tryptophan residues are replaced with 5-fluorotryptophan. The resulting ^19^F spectrum shows five separated resonances of which we assigned the most upfield signal to W43 through mutagenesis (Figs. 4A, S15). The ^19^F resonance of W43 in the NRD thus shifted significantly downfield due to the interaction with the CTD. This confirms that Dcp2 is mostly present in the closed conformation. To assess this finding independently, we prepared a sample that contains ^19^F labeled tryptophan residues as well as methyl ^13^C labeled isoleucine and methionine residues. Previously, we showed that the resonance frequencies of M164 and M221 in methyl TROSY spectra directly report on the open-closed equilibrium in Dcp2 (Wurm et al. 2017). Importantly, the incorporation of ^19^F tryptophan only had a very small effect on the position of those resonances (Fig. 4B) that corresponds to a less than 5% shift of the Dcp2 conformational equilibrium towards the closed state. This finding corroborates that the introduction of 5-fluorotryptophan has, if at all, a limited impact on the structure and dynamics of protein complexes (Fig. 4B). Based on the ^19^F 1D spectra of the 46 kDa Dcp1:Dcp2 complex we subsequently attempted to record ^19^F based CPMG experiments. We realized, however, that the ^19^F R_2_ relaxation rates were around 200 s^−1^, which prevents the use of sufficiently long CPMG relaxation delays that are required for the recording of CPMG frequencies lower than a couple of 100 Hz. In that light, the Dcp1:Dcp2 complex that contains both the NRD and the CTD is beyond the limit of where 5-fluorotryptophan based ^19^F spectroscopy can be employed to extract detailed information regarding protein dynamics. It should be noted that the molecular weight of the Dcp1:Dcp2-NRD-CTD enzyme complex also prevented us from assessing dynamics through ^15^N based RD experiments. We have, however, shown that high quality ^19^F RD CPMG curves can be recorded for the 100 kDa Xrn2 enzyme after labeling the protein with BTFA (Overbeck et al. 2022). This shows that the fluorine relaxation properties of 5-fluorotryptophan residues are less advantageous than those of CF_3_ groups on cysteine side-chains.

### DcpS scavenger decapping complex

**Fig. 5:**
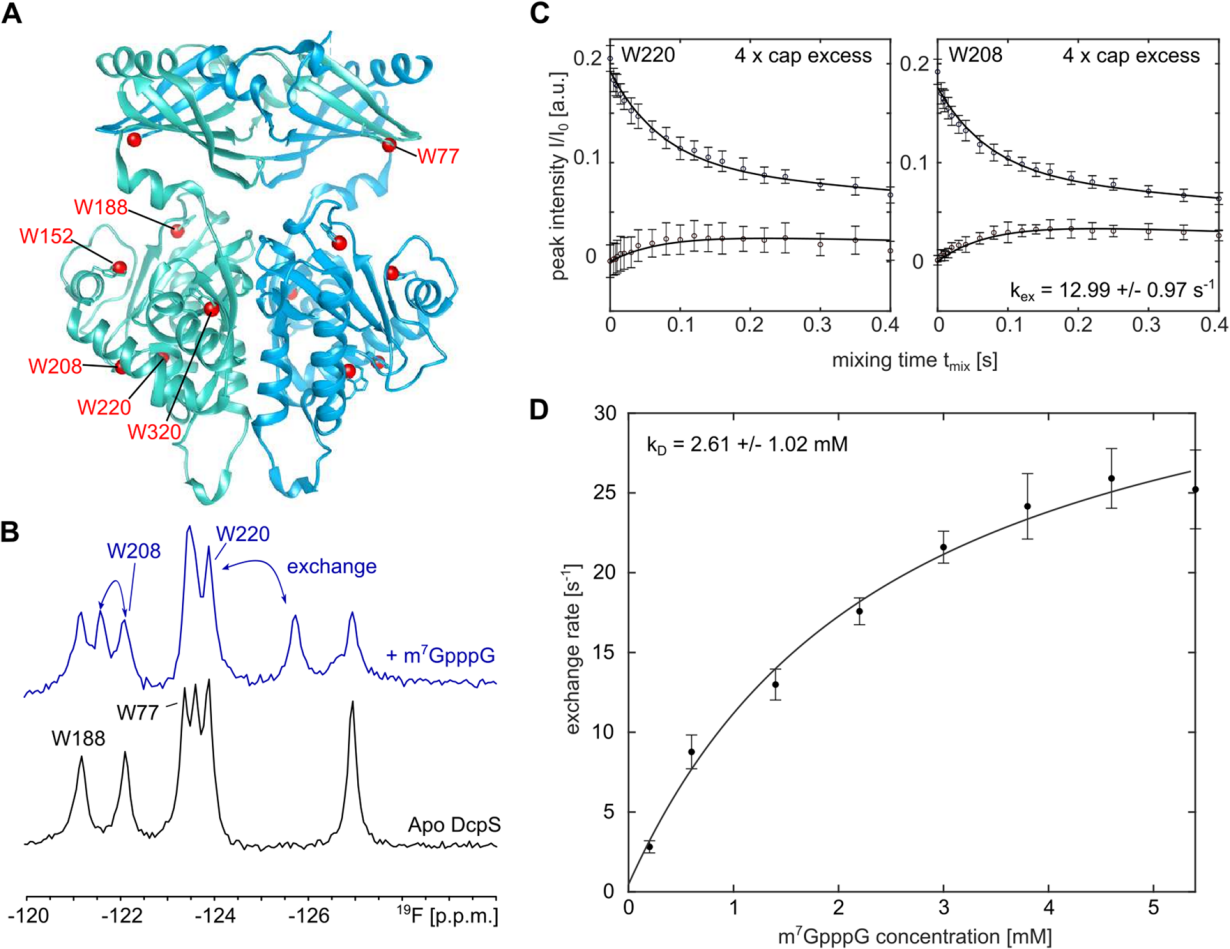
The 80 kDa DcpS scavenger decapping enzyme. **A** Structure of the homodimeric enzyme, with the two different protomers colored in green and blue. The seven tryptophan residues are highlighted as red spheres. **B** ^19^F NMR spectra of the apo enzyme (bottom) and the enzyme in complex with m^7^GpppG (1 substrate per dimer). Assignments for the fluorine resonances are indicated. **C** Longitudinal exchange experiment to quantify the flipping motion in the enzyme in the presence of a fourfold molar excess of the m^7^GpppG substrate. The two panels visualize the exchange that is sensed by W208 and W220, the drawn line corresponds to a global fit of the data to a two-site exchange mechanism, where the populations of state A and B are fixed to 0.5. **D** The flipping rate increases with increasing substrate concentration. The drawn line corresponds to a fit of the data to a two state binding model of the DcpS enzyme and the m^7^GpppG ligand

Finally, we assessed if ^19^F NMR methods can accurately address motions in the 80 kDa scavenger decapping enzyme DcpS. DcpS is a homo-dimer that contains two active sites at the interface between the N- and C-terminal lobes of the enzyme (Fig. 5A) (Gu et al. 2004). Upon recruitment of a short mRNA substrate, the N- and C-terminal lobes close around one of the two active sites, which simultaneously opens the other active site. As a result, the complex transits from a symmetric apo conformation to an asymmetric substrate bound conformation. Binding of a second substrate to the open binding site of the asymmetric DcpS:substrate complex results in seesaw-like flipping motions, where the two active sites on both sides of the enzyme alternatingly close and open. Previously, we have shown that the rate of these flipping motions increases with increasing substrate concentration (Neu et al. 2015; Fuchs et al. 2020). For our studies here, we used the DcpS enzyme from *C. thermophilum* that contains six tryptophan residues and that yields high quality ^19^F NMR spectra (Fig. 5B). Of note, the ^19^F NMR spectra of the human and the *S. cerevisiae* DcpS enzymes yielded ^19^F spectra of inferior quality, rendering those unsuitable for detailed ^19^F NMR experiments (Fig. S16). Previously, we reported that the seesaw motions in DcpS take place at rates that are too slow to be accurately captured by CPMG and/or R_1ρ_ experiments. Here, we thus turned to longitudinal EXSY experiments, where we determine the intensity of cross peaks in a series of 2D NOESY spectra that were recorded with different mixing times. This is possible in the current case as all ^19^F-^19^F distances in the enzyme are over 15 Å (Fig. 5A) and all cross peaks in the spectrum must thus result from exchange and not from NOEs. Upon addition of an equimolar amount of the short m^7^GpppG mRNA cap substrate (1 substrate per DcpS monomer), a number of the resonances split, as a result of the formation of the asymmetric conformation in DcpS (Fig. 5B). Based on the decay of the auto peaks and the buildup of exchange peaks in the EXSY spectra (Fig. 5C) we extracted an exchange rate of 2.8 ± 0.4 s^−1^ for the seesaw like motion in DcpS. We subsequently added 2-, 4-, 6-, 8-, 10-, 12- and 14-fold molar excesses of the mRNA cap substrate and observed a step-wise increase in the exchange rate to 25.2 ± 2.5 s^−1^ (Figs. 5D, S17). This dependency of the rate of the seesaw motion on the substrate concentration agrees with previous studies that we performed on the *S. cerevisiae* DcpS enzyme (Neu et al. 2015), where we made use of methyl TROSY spectroscopy on deuterated and isoleucine labeled samples (Schütz and Sprangers 2019). Based on that, we conclude that ^19^F NMR is, in this case, a suitable alternative to methyl TROSY NMR spectroscopy for the quantification of conformational exchange in the presence of varying amounts of substrate. Mechanistically, the addition of increasing amounts of ligand results in the increased occupation of the open binding site. This notion is affirmed by the observation that the exchange rates at the different ligand concentrations can be fitted to a simple binding model (Fig. 5D). Based on that, an affinity of m^7^GpppG for the open binding site of 2.6 ± 1.0 mM can be extracted. This affinity agrees well with the affinity that is extracted from the shift of the fluorine resonance in the open binding site upon substrate addition (2.48 ± 0.85 mM) (Fig. S18). Binding of the m^7^GpppG ligand and the rate of the DcpS flipping motions are thus directly correlated.

## Discussion

Protein dynamics are essential for function. It is thus important to have access to methods that can determine the thermodynamic and kinetic parameters that are associated with protein motions. Solution state NMR spectroscopy has proven to be a highly successful method in that regard. Most NMR methods rely on labeling strategies that visualize the protein backbone through ^1^H-^15^N based spectra or on the use of methyl containing side chains in combination with ^1^H-^13^C methyl TROSY based experiments. These methods provide information with a high spatial resolution and relaxation data is normally recorded as a set of 2D experiments. In that regard, ^19^F NMR methods can have an advantage as these can often be recorded rapidly in a 1D manner due to the limited number of well resolved resonances.

Numerous impressive examples are available in the literature where ^19^F NMR methods have been exploited to extract information regarding protein motions (Kitevski-LeBlanc and Prosser 2012). These include studies of a fold-switch in the mycobacterial glycosyltransferase PimA (Liebau et al. 2020), calmodulin (Hoang and Prosser 2014), the nonstructural protein 1 of influenza A virus (Aramini et al. 2014), GPCRs (Liu et al. 2012; Kim et al. 2013; Didenko et al. 2013; Manglik et al. 2015; Frei et al. 2020; Pan et al. 2022), the periplasmic glucose/galactose receptor (Luck et al. 1996), diacyl-glycerol kinase (Shi et al. 2011), a cold shock protein (Overbeck et al. 2020), alkaline phosphatase (Hull and Sykes 1974), the chaperone PapD (Bann et al. 2002), spider silk (Sarker et al. 2016), the protease trypsin (Ruben et al. 2020) and the Xrn2 exoribonuclease (Overbeck et al. 2022). However, to our knowledge, it has never been systematically evaluated whether the incorporation of ^19^F labels has an effect on the native protein dynamics, despite reports that show that ^19^F labeling can have an effect on protein structure and stability (Xiao et al. 1998; Minks et al. 1999; Acchione et al. 2012; Dalvit and Vulpetti 2016).

Here, we show that the incorporation of 5-fluorotryptophan residues has, if at all, a minor effect on the native dynamics in a number of globular proteins (the KIX domain, Dcp1 or Dcp2). This result is encouraging and a prerequisite for the use of ^19^F NMR methods. This finding is also in agreement with previous reports that showed that the overall structure of the GB1 protein is not influenced by the incorporation of 5- fluorotryptophan (Campos-Olivas et al. 2002) and that the stability of the cold shock protein B from *B. subtilis* is unaffected by fluorine labeling (Welte et al. 2020).

Proteins are dynamic at multiple timescales at the same time. The lifetimes of the states correlate with the energy barrier between the states, which is normally small for local motions of loops and side-chains (faster timescale motions) and significantly higher for global concerted motions (slower timescale motions). Slower timescale motions in enzymes have been directly linked with function and studying these motions is thus of a high biological interest. Fast side-chain motions are also functionally important and have been correlated with the conformational entropy of proteins, which plays a role in e.g. protein stability and ligand interactions (Kasinath et al. 2013; Wand and Sharp 2018; Hoffmann et al. 2022). The CPMG RD experiments that we record here reveal dynamics of the incorporated 5-fluorotryptophan residues with exchange rates that are on the order of 10,000 s^−1^. These fast motions could originate from rotameric jumps of the aromatic side-chains. Interestingly, these fast side-chain motions are readily detected in ^19^F based experiments, but were not visible in nitrogen based CPMG RD experiments on the H_ε_-N_ε_ groups of the indole rings. This difference between the fluorine and nitrogen based RD experiments results from the lower gyromagnetic ratio (γ_15N_ ~ 0.11* γ_19F_) and the smaller chemical shift range of nitrogen. As a result the |Δω_N_| values are considerably smaller than the corresponding |Δω_F_| values. When the local side chain motions are relatively fast, as we observe here, the amplitude of the RD curves depends on |Δω|^2^. Consequently, the amplitude of the nitrogen based RD curves will be significantly smaller and cannot be detected reliably anymore. Slower motions can, however, be well detected using nitrogen based RD experiments (Figs. 1, 2, 3). ^19^F based RD experiments are also highly sensitive to the motions that we detected in the backbones of the KIX domain, Dcp1 and Dcp2. The highly sensitive ^19^F experiments will thus simultaneously detect faster (side chain) and slower (backbone) motions of the 5-fluorotryptophan residues. Depending on the rates, population and chemical shift differences the RD curves can be strongly biased towards the faster of the slower of the two processes and the resulting RD curves cannot be analyzed using a two-state model anymore. As it is a-priory not possible to conclude if multiple exchange processes are superimposed or not, we suggest to support kinetic and thermodynamic properties that are derived from ^19^F measurements with complementary data. This is especially relevant when the ^19^F RD curves indicate the presence of faster motions that most likely result from side chain dynamics and not from global motions. In that light it is worth mentioning that we previously validated ^19^F derived parameters for the Xrn2 enzyme with data that was recorded on methyl groups (Overbeck et al. 2022). Likewise, we here show that EXSY experiments, that are insensitive to potential tryptophan side chain motions that are fast on the NMR chemical shift timescale, are very well suited to extract slow processes like the domain flipping motions in DcpS. Based on that, we anticipate that a careful interpretation of fluorine relaxation data will result in ample insights into biological function.

## Supplementary Information

Below is the link to the electronic supplementary material.

Supplementary file1 (PDF 4191 kb)
