# Supplementary material for: Assessing the applicability of 19F labeled tryptophan residues to quantify protein dynamics
Source: J Biomol NMR. 2023 Jan 14;77(1-2):55–67. doi: 10.1007/s10858-022-00411-2 (PMC10149471; doi:10.1007/s10858-022-00411-2)
Supplement: Supplementary file 1 — Supplementary file1 (PDF 4191 kb) [file 10858_2022_411_MOESM1_ESM.pdf]

Supplementary Information

Assessing the applicability of ^{19}F labeled tryptophan residues to quantify protein dynamics.

Authors:

Christina Krempl [a] and Remco Sprangers*[a]

[a] Department of Biophysics I, Regensburg Center for Biochemistry, University of Regensburg, 93053 Regensburg, Germany.

* Correspondence to: Remco Sprangers, Department of Biophysics I, Regensburg Center for Biochemistry, University of Regensburg, 93053 Regensburg, Germany.

E-mail: remco.sprangers@ur.de

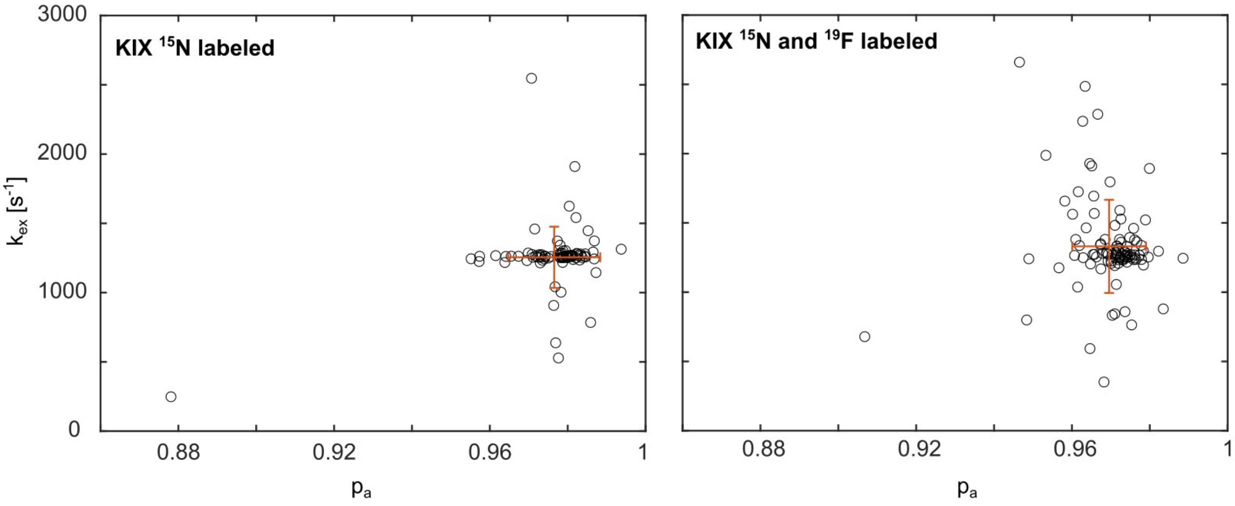

Fig. S1 Result of 100 Monte Carlo simulations used to extract exchange parameters for the ^{15}N CPMG RD data recorded on the ^{15}N labeled KIX domain (left; $p_a = 97.7 \pm 1.2$ %, $k_{ex} = 1255 \pm 221$ s^{-1}) and on the ^{15}N 5-fluorotryptophan labeled KIX domain (right; $p_a = 97.0 \pm 1.0$ %, $k_{ex} = 1331 \pm 336$ s^{-1}). The orange cross represents the average population of the ground state (p_A) and exchange rate (k_{ex}). The error bars indicate the standard deviations in both parameters.

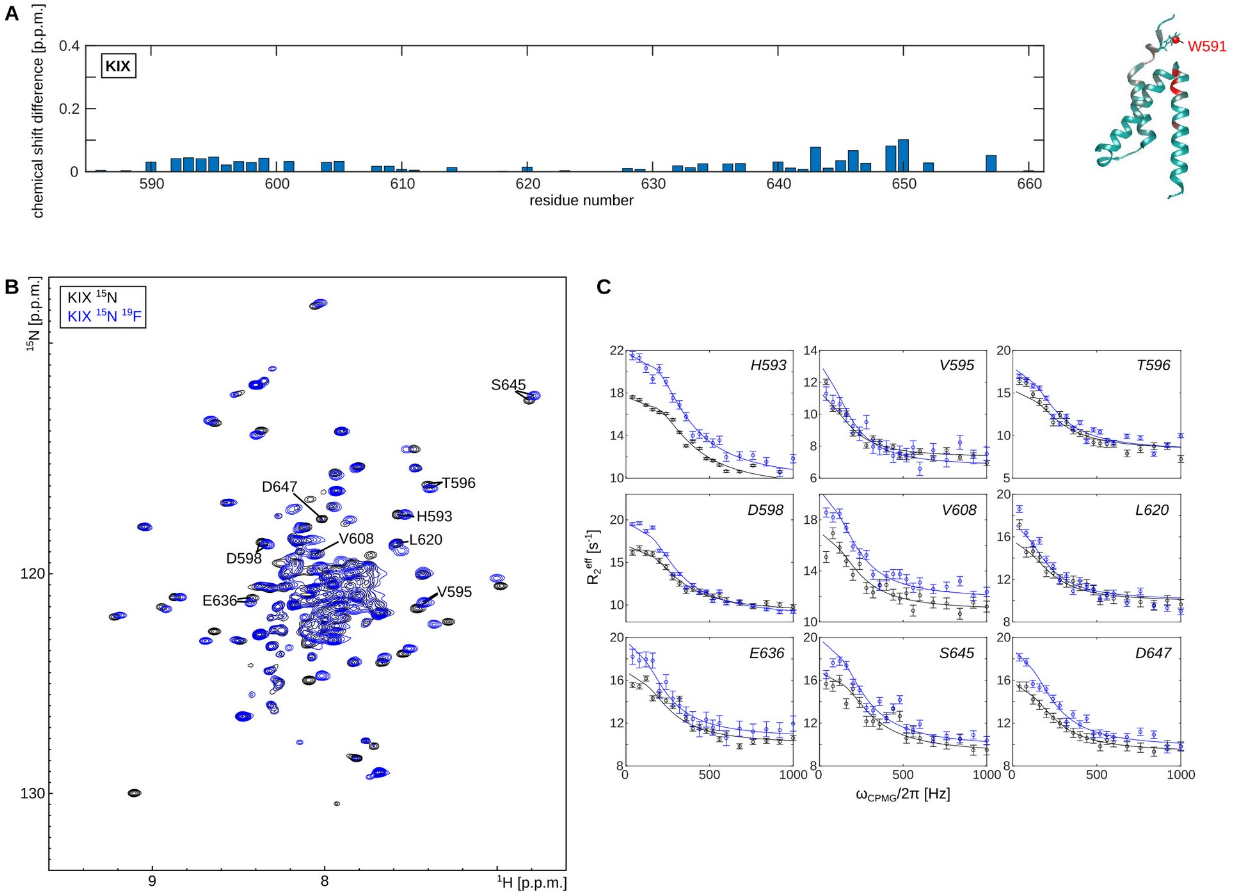

Fig. S2 Differences in the NMR spectra and CPMG RD curves of the ^{15}N and ^{15}N ^{19}F labeled KIX domain.

(A) Chemical shift perturbations in the KIX domain upon replacement of all tryptophan residues with 5-fluorotryptophan. CSPs are calculated in ppm as $CSP = \sqrt{\left((0.1 * \Delta\delta N)^2 + (\Delta\delta H)^2\right)}$. The structures on the right are colored according to the CSPs, where the replaced tryptophan residues are shown as sticks.

(B) Overlay of the ^{1}H-^{15}N spectra of the ^{15}N (black; Figure 1A) and ^{15}N ^{19}F (blue; Figure 1C) labeled KIX domain.

(C) Overlay of the ^{15}N CPMG RD curves recorded on the ^{15}N (black; Figure 1B) and ^{15}N ^{19}F (blue; Figure 1D) labeled KIX domain. The differences in the amplitude of the RD curves can be attributed to changes in the chemical shift differences ($|\Delta\omega_N|$) between the ground and excited state between the two labeling schemes and do not necessarily reflect differences in populations or exchange rates between the two samples.

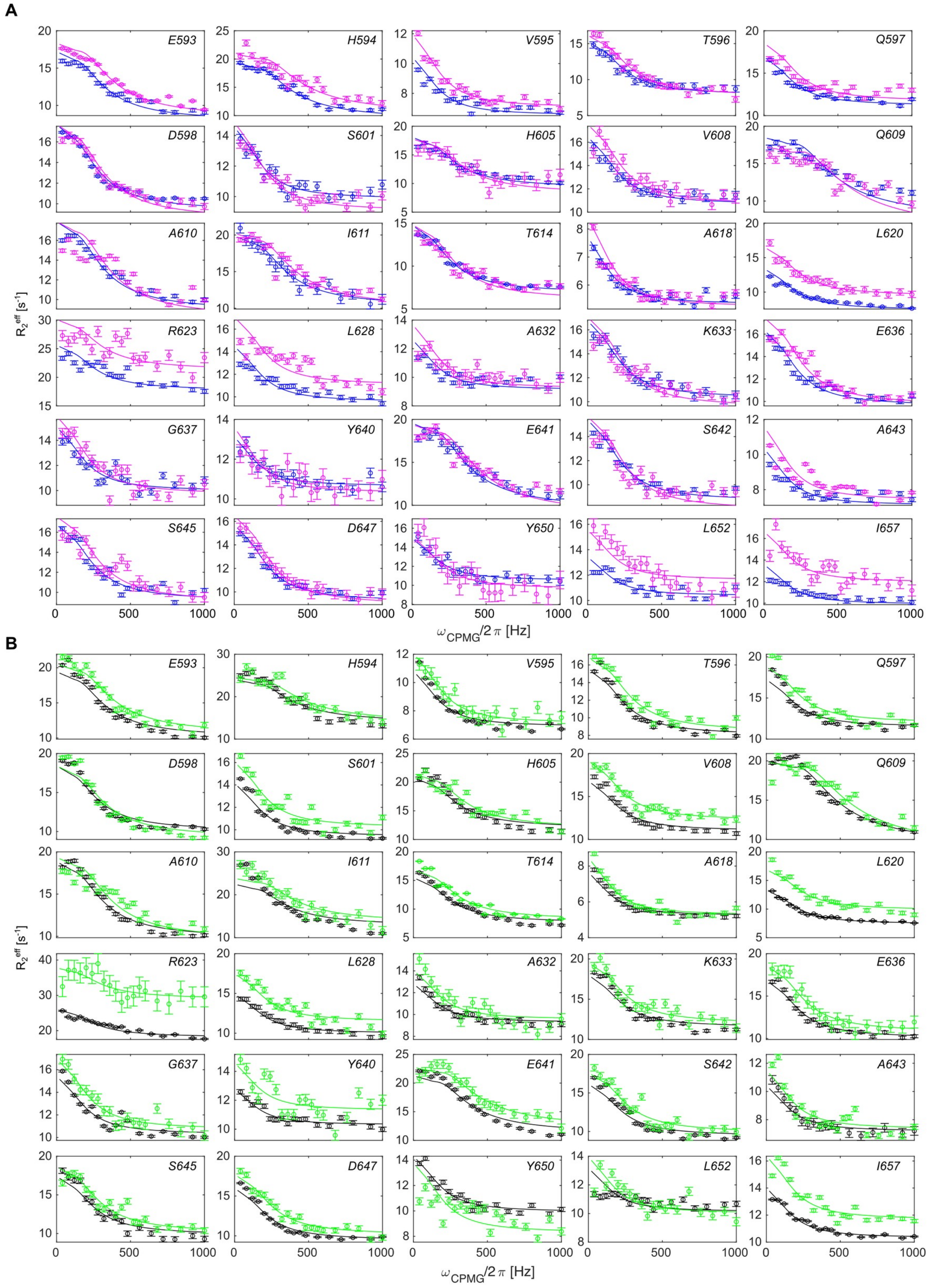

Fig. S3 ^{15}N relaxation dispersion profiles of the ^{15}N (A) and ^{15}N 5-fluorotryptophan labeled KIX domain (B) recorded at a 500 MHz proton frequency (blue, black) and 600 MHz proton frequency

(pink, green). The drawn lines are a simultaneous fit of all data from both proteins to a two-site exchange process (p_a = 98.2 ± 0.4 %; k_{ex} = 924 ± 136 s^{-1}).

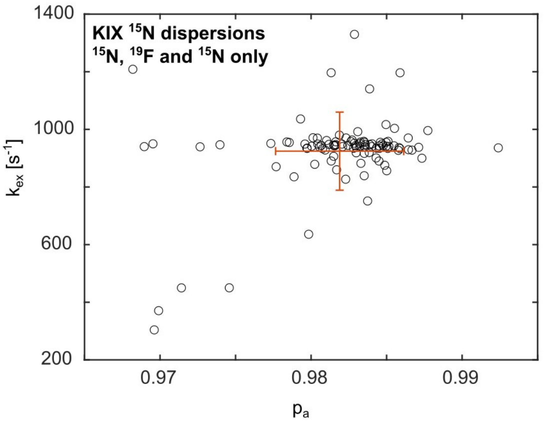

Fig. S4 Result of 100 Monte Carlo simulations to extract exchange parameters for a simultaneous fit of the ^{15}N CPMG RD data recorded on the ^{15}N labeled KIX domain and on the ^{15}N 5-fluorotryptophan labeled KIX domain (p_a = 98.2 ± 0.4 %; k_{ex} = 924 ± 136 s^{-1}). The orange cross represents the average population of the ground state (p_A) and exchange rate (k_{ex}). The error bars indicate the standard deviations in both parameters.

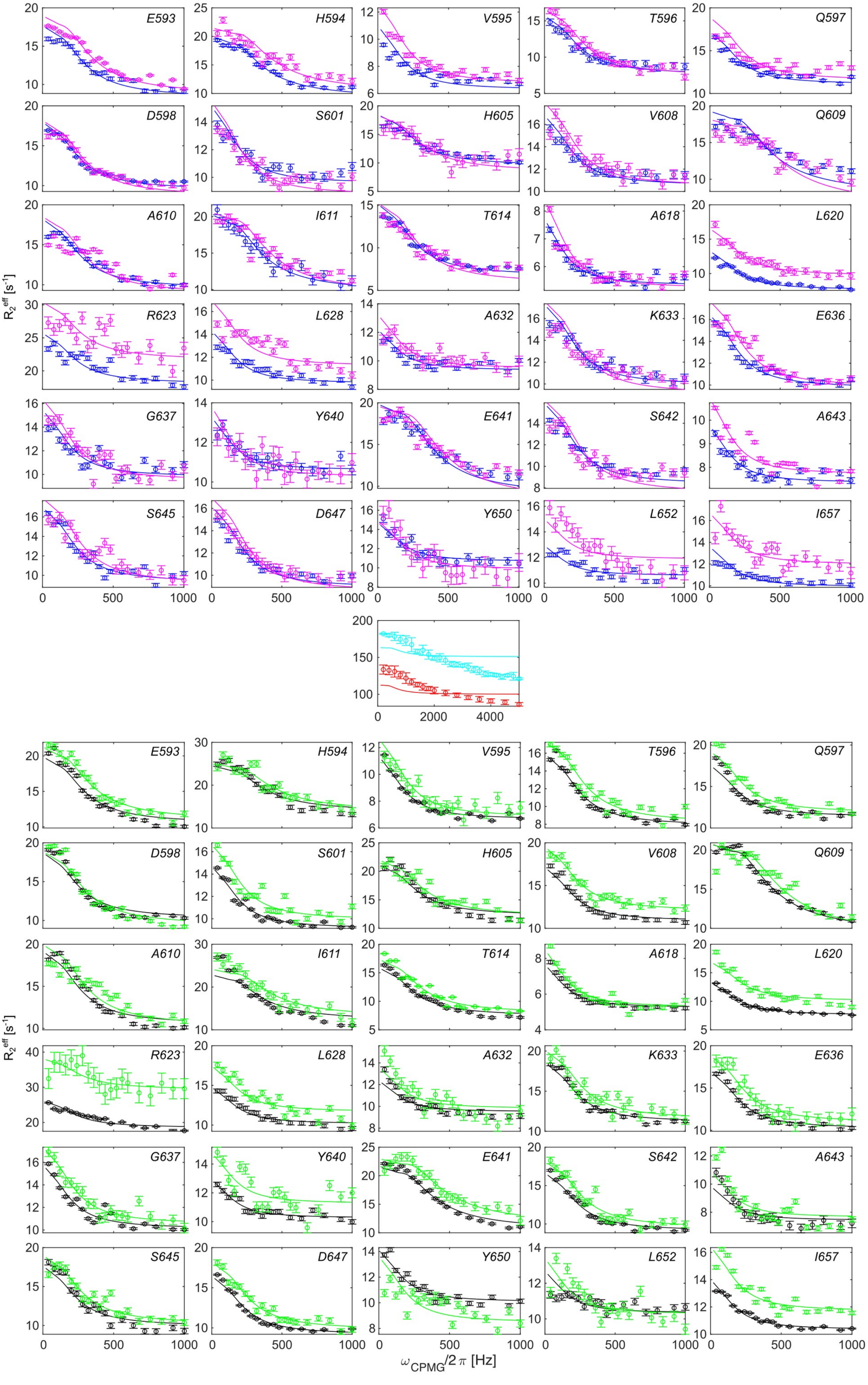

E593
H594
V595
T596
Q597
D598
S601
H605
V608
Q609
A610
I611
T614
A618
L620
R623
L628
A632
K633
E636
G637
Y640
E641
S642
A643
S645
D647
Y650
L652
I657
R_2^{eff} [s^{-1}]
$\omega_{CPMG}/2\pi$ [Hz]

Fig. S5 Relaxation dispersion (RD) profiles of the ^{15}N labeled KIX domain (top; ^{15}N RD) and the ^{19}F and ^{15}N labeled KIX domain (middle; ^{19}F RD and bottom; ^{15}N RD). Data was recorded at a 500 MHz proton frequency (blue, red, black) and 600 MHz proton frequency (pink, cyan, green). The drawn lines represent an effort to simultaneously fit all data from both proteins to a two-site exchange process. The ^{19}F and ^{15}N data are not compatible (see middle panel), indicating that the RD data of the 5-fluorotryptophan and the backbone are not the same.

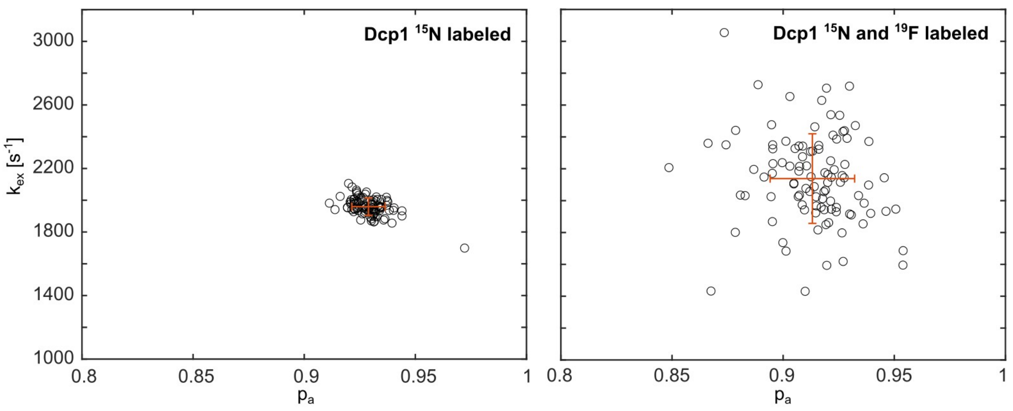

Fig. S6 Result of Monte Carlo to extract exchange parameters for the ^{15}N CPMG RD data recorded on the ^{15}N labeled Dcp1 protein (left) and on the ^{15}N 5-fluorotryptophan labeled Dcp1 protein (right). The orange cross represents the average population of the ground state (p_A) and exchange rate (k_{ex}). The error bars indicate the standard deviations in both parameters.

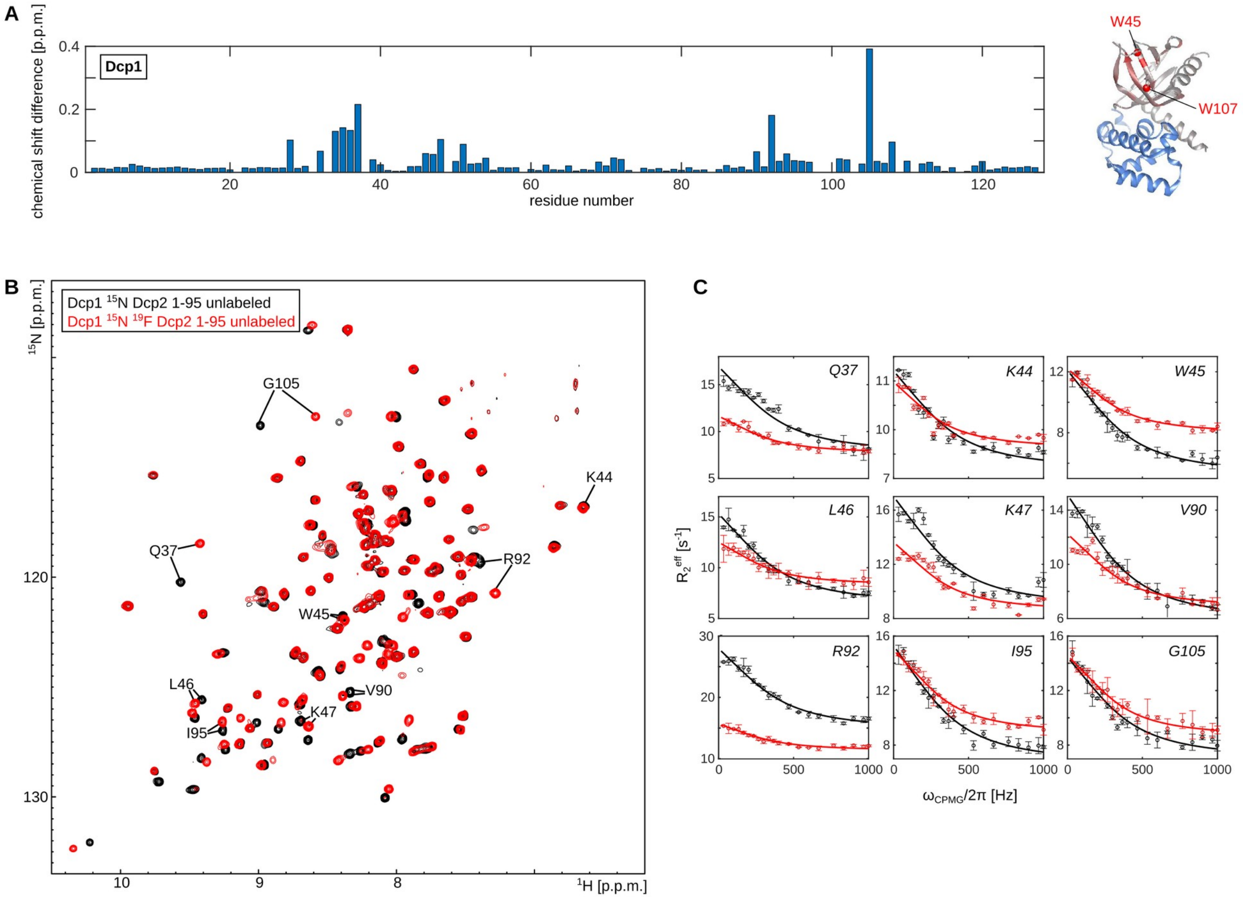

Fig. S7 Differences in the NMR spectra and CPMG RD curves of ^{15}N and ^{15}N ^{19}F labeled Dcp1 in the Dcp1:Dcp2 complex.

(A) Chemical shift perturbations in Dcp1 upon replacement of all tryptophan residues with 5-fluorotryptophan. CSPs are calculated in ppm as $CSP=\sqrt{((0.1*\Delta\delta N)^2+(\Delta\delta H)^2)}$. The structures on the right are colored according to the CSPs, where the replaced tryptophan residues are shown as sticks.

(B) Overlay of the ^{1}H-^{15}N spectra of ^{15}N (black; Figure 2A) and ^{15}N ^{19}F (red; Figure 2C) labeled Dcp1.

(C) Overlay of the ^{15}N CPMG RD curves recorded on ^{15}N (black; Figure 2B) and ^{15}N ^{19}F (red; Figure 2D) labeled Dcp1. The differences in the amplitude of the RD curves can be attributed to changes in the chemical shift differences ($|\Delta\omega_N|$) between the ground and excited state between the two labeling schemes and do not necessarily reflect differences in populations or exchange rates between the two samples.

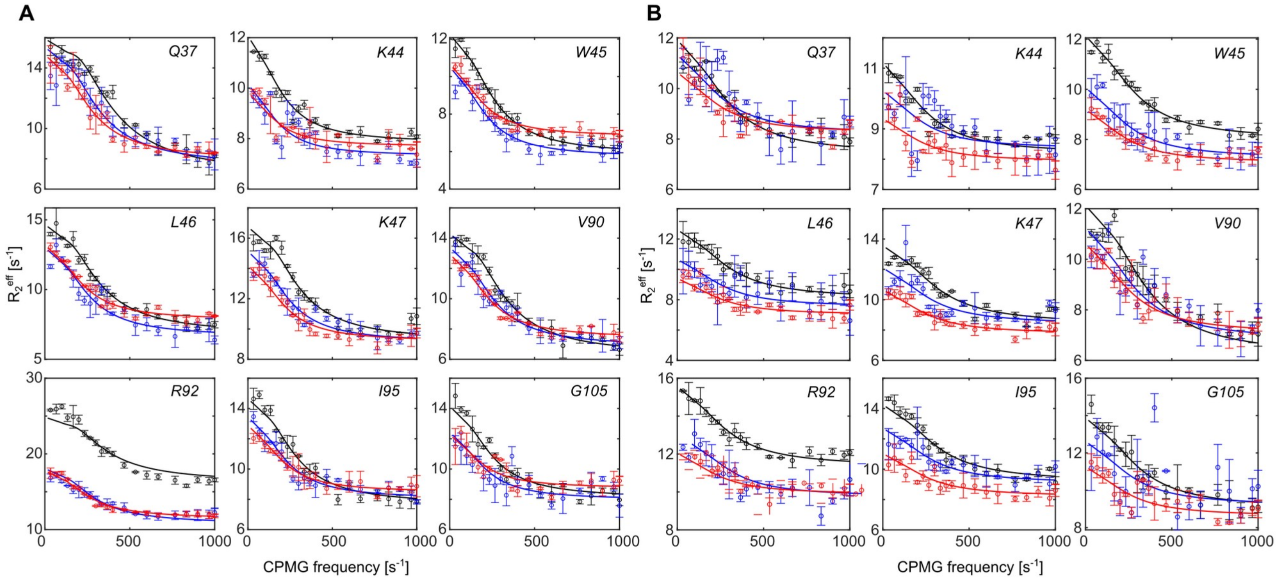

Fig. S8 ^{15}N relaxation dispersion profiles of the ^{15}N (A) and ^{15}N 5-fluorotryptophan labeled Dcp1 protein (B) recorded at a 500 MHz (red), 600 MHz (blue) and 800 (black) MHz proton frequency. The drawn lines are a simultaneous fit of all data from both proteins to a two-site exchange process (p_a = 91.8 ± 1.1 %; k_{ex} = 2220 ± 121 s^{-1}).

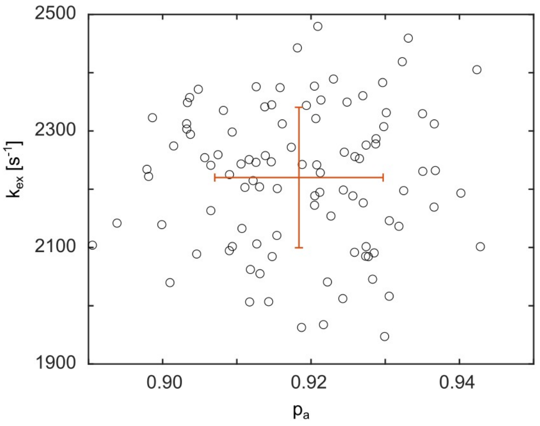

Fig. S9 Result of 100 Monte Carlo simulations to extract exchange parameters for a simultaneous fit of the ^{15}N CPMG RD data recorded on the ^{15}N labeled Dcp1 protein and on the ^{15}N 5-fluorotryptophan labeled Dcp1 protein. The orange cross represents the average population of the ground state (p_A) and the exchange rate (k_{ex}). The error bars indicate the standard deviations in both parameters.

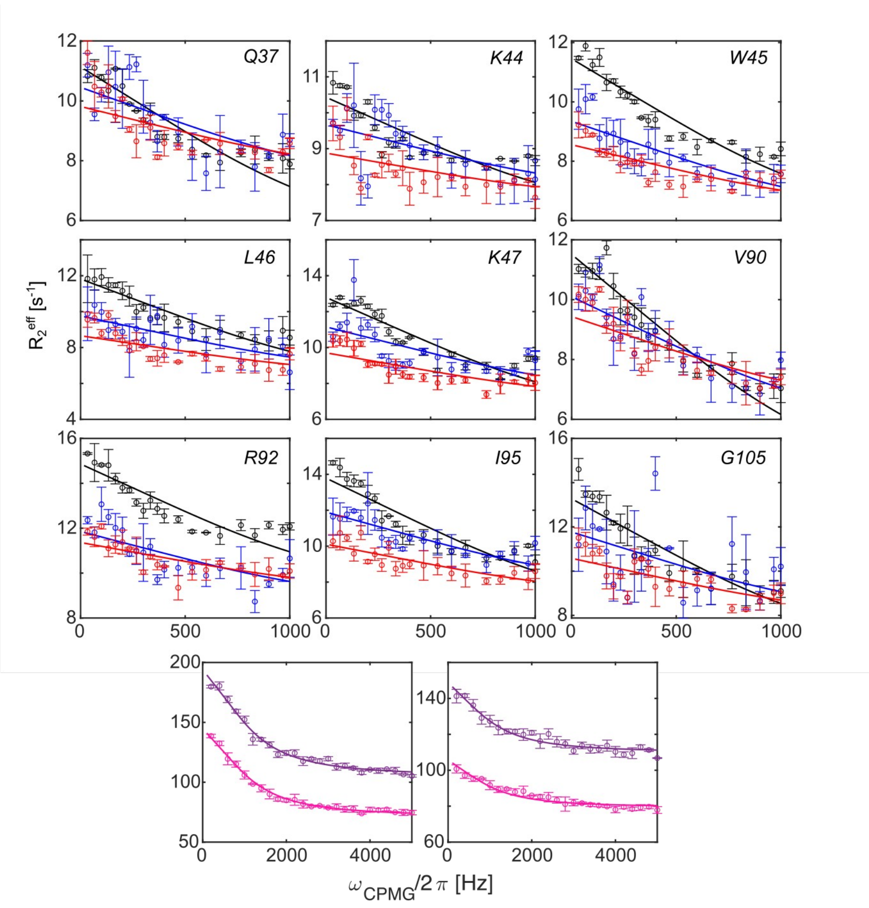

Fig. S10 ^{15}N (top) and ^{19}F (bottom) relaxation dispersion profiles recorded on the ^{15}N, 5-fluorotryptophan labeled Dcp1 protein. ^{15}N data was recorded at a 500 MHz (red), 600 MHz (blue) and 800 (black) MHz proton frequency, ^{19}F data was recorded at 500 (pink) and 600 (purple) MHz protein frequency. The drawn lines represent an effort to simultaneously fit the ^{15}N and ^{19}F RD data to a two-site exchange process. The ^{19}F and ^{15}N data are not compatible, indicating that the nitrogen and fluorine RD profiles do not report on the same process.

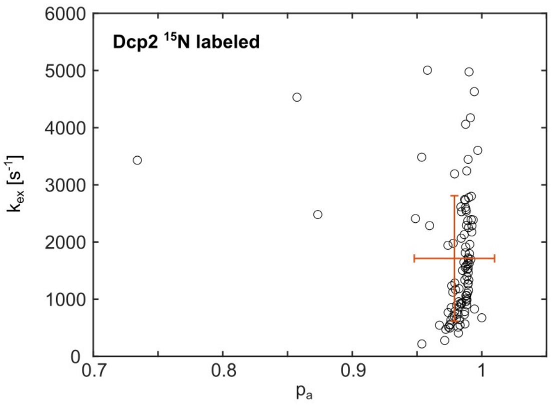

Fig. S11 Result of Monte Carlo to extract exchange parameters for the ^{15}N CPMG RD data recorded on the ^{15}N 5-fluorotryptophan labeled Dcp2 protein in the Dcp1:Dcp2 NTD complex. The red cross represents the average population of the ground state (p_A) and exchange rate (k_{ex}). The error bars indicate the standard deviations in both parameters. $p_a = 97.9 \pm 3.1$ %; $k_{ex} = 1712 \pm 1098$ s^{-1}.

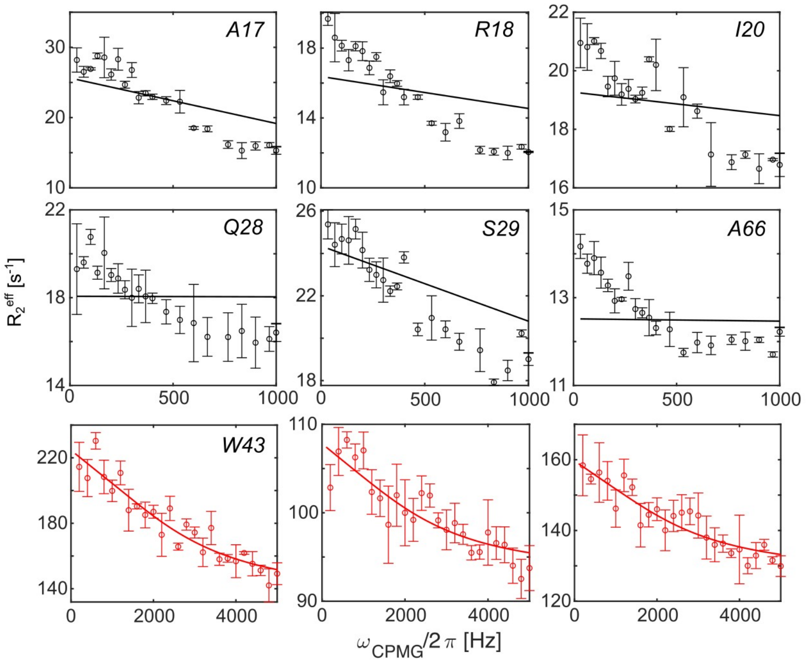

Fig. S12 ^{15}N (black) and ^{19}F (red) relaxation dispersion profiles recorded on the ^{15}N, 5-fluorotryptophan labeled Dcp2 protein. ^{15}N data was recorded at 800 (black) MHz proton frequency, ^{19}F data was recorded at 600 (red) MHz protein frequency. The drawn lines represent an effort to simultaneously fit the ^{15}N and ^{19}F RD data to a two-site exchange process. The ^{19}F and ^{15}N data are not compatible, indicating that the motions of the 5-fluorotryptophan and the motions of the backbone are not the same.

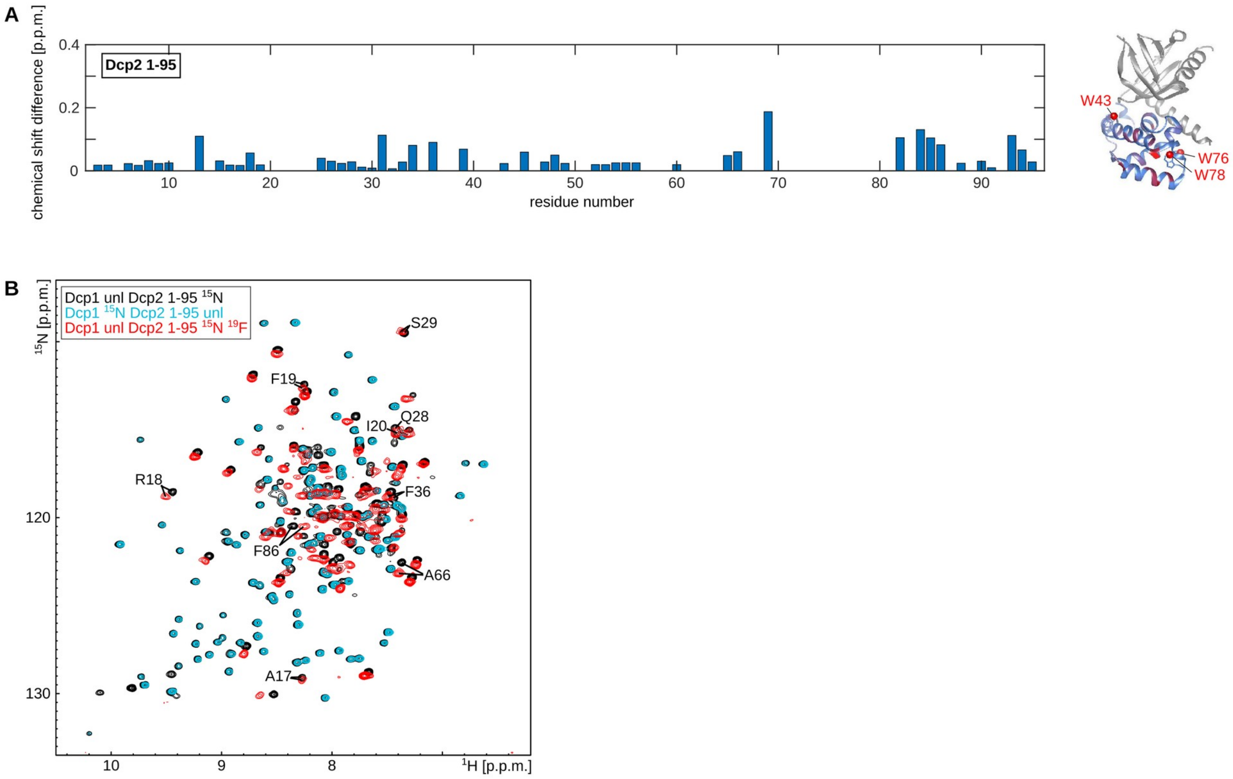

Fig. S13 Differences in the NMR spectra of ^{15}N and ^{15}N ^{19}F labeled Dcp2 in the Dcp1:Dcp2 complex.

(A) Chemical shift perturbations in Dcp2 upon replacement of all tryptophan residues with 5-fluorotryptophan. CSPs are calculated in ppm as $CSP=\sqrt{((0.1*\Delta\delta N)^2+(\Delta\delta H)^2)}$. The structures on the right are colored according to the CSPs, where the replaced tryptophan residues are shown as sticks.

(B) Overlay of the ^{1}H-^{15}N spectra of the ^{15}N labeled Dcp1:Dcp2 complex (black; both Dcp1 and Dcp2 are ^{15}N labeled), the Dcp1:Dcp2 complex where Dcp2 is ^{15}N ^{19}F labeled (red) and the Dcp1:Dcp2 complex in which Dcp1 is ^{15}N labeled (blue).

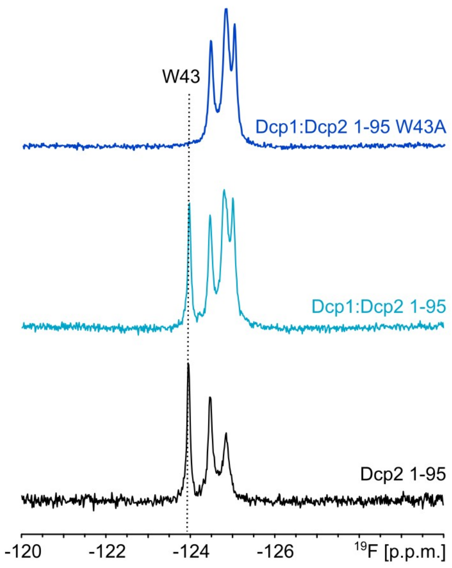

Fig. S14 Assignment of the W43 ^{19}F 5-fluorotryptophan resonance in Dcp2 (residues 1-95). The lower (black) spectrum shows the resonances of the three Dcp2 Trp residues in the Dcp1:Dcp2 (residues 1-95) complex (Dcp1 is not ^{19}F labeled). The middle (light blue) spectrum shows the resonances of the five Trp resonances in the Dcp1:Dcp2 (residue 1-95) complex. The top spectrum shows the resonances of the four Trp resonances in the Dcp1:Dcp2 (residue 1-95; W43A) complex. The most upfield shifted signal disappears, allowing a straightforward assignment of this resonance to W43.

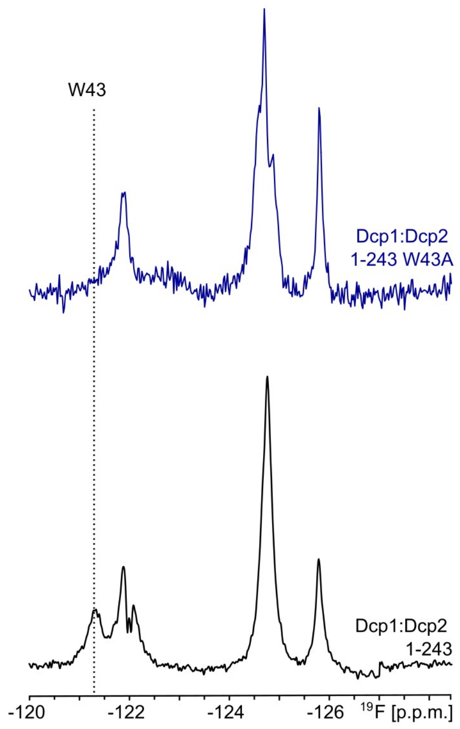

Fig. S15 Assignment of the W43 ^{19}F 5-fluorotryptophan resonance in Dcp2 (residues 1-243). The lower (black) spectrum shows the resonances of the seven Dcp2 Trp residues in the Dcp1:Dcp2 (residues 1-243) complex (Dcp1 is not ^{19}F labeled). The top (blue) spectrum shows resonances of the six Trp resonances in the complex where tryptophan 43 is replaced with an alanine. The most upfield signal disappears, allowing the assignment of this resonance to W43. Note that the frequency of W43 shifts from -123.95 (Fig. S11) to -121.31 ppm when the Dcp2 catalytic domain is included in the Dcp1:Dcp2 complex.

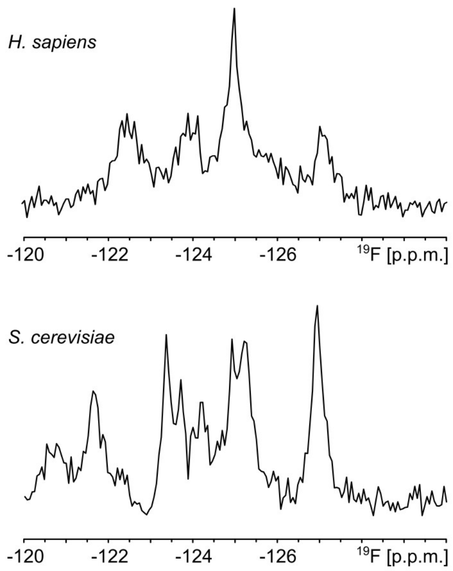

Fig. S16 ^{19}F NMR spectra (500 MHz proton frequency) of the 80 kDa DcpS complexes from *H. sapiens* (245 µM; top) and from *S. cerevisiae* (165 µM; bottom). The spectral quality is low, preventing accurate extraction of protein dynamics.

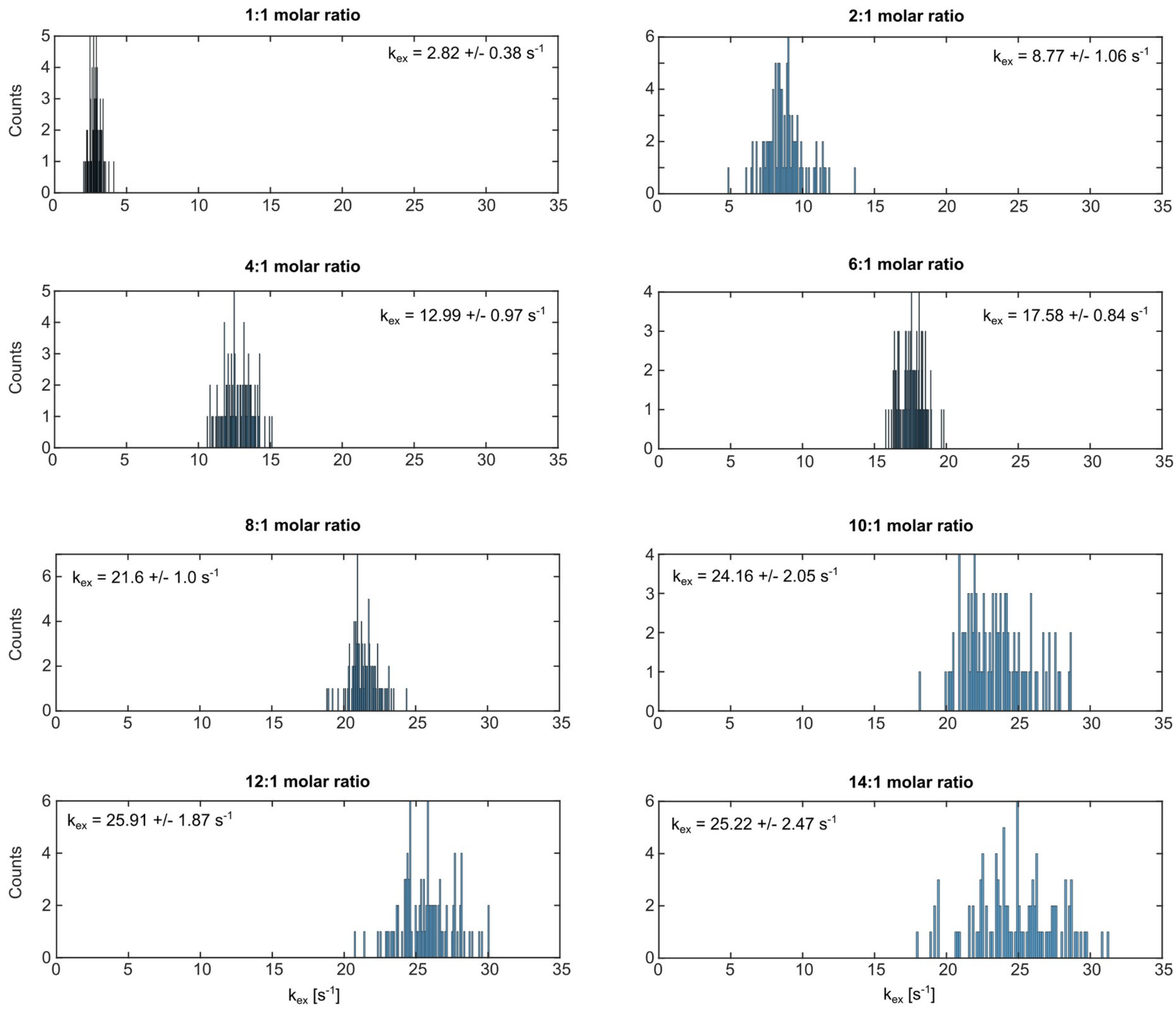

Fig. S17 DcpS flipping rates extracted from 100 Monte Carlo simulations at different substrate: enzyme ratios.

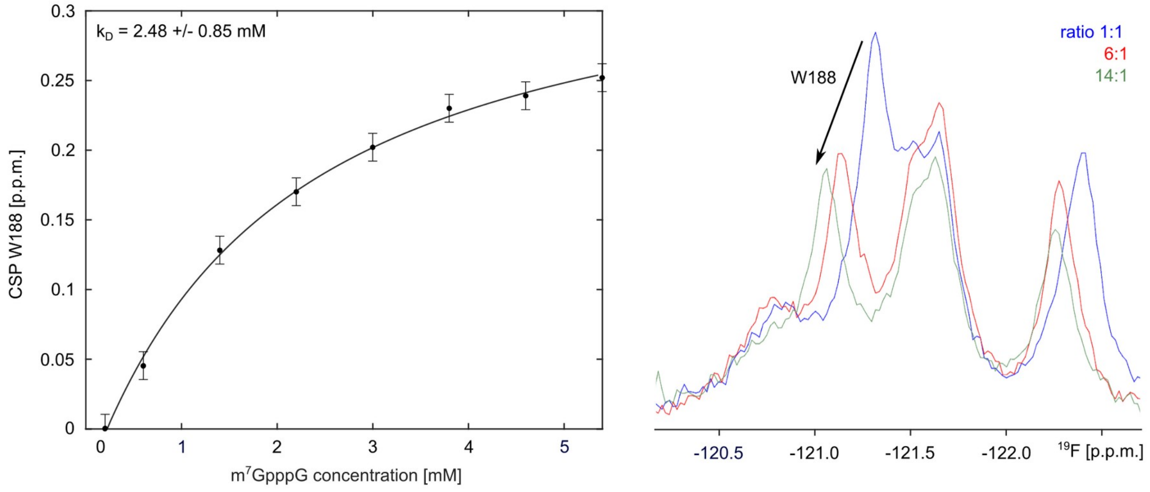

Fig. S18 Determination of the DcpS:m^{7}GpppG affinity based on the shift of one of the Trp resonances with increasing amounts of the ligand.
